# Prenatal to early postnatal neurotrophic treatment prevents Alzheimer-like behavior and pathology in mice

**DOI:** 10.1186/s13195-020-00666-7

**Published:** 2020-08-27

**Authors:** Wei Wei, Yifan Wang, Yinghua Liu, Chun-Ling Dai, Yunn-Chyn Tung, Fei Liu, Khalid Iqbal

**Affiliations:** 1 Department of Neurochemistry, Inge Grundke-Iqbal Research Floor, New York State Institute for Basic Research in Developmental Disabilities, Staten Island, New York, USA; 2 Key Laboratory of State Administration of Traditional Chinese Medicine of China, Department of Pathophysiology, School of Medicine, Institute of Brain Research, Jinan University, Guangzhou, China; 3 Department of Pharmacology, School of Pharmaceutical Sciences, Guangzhou Medical University, Guangzhou, China

**Keywords:** Alzheimer’s disease, Ciliary neurotrophic factor (CNTF)–derived peptide, Cognition, Amyloid-β, Tau pathology, Neuroplasticity, Neuroinflammation

## Abstract

**Background:**

Alzheimer’s disease (AD) is a progressive neurodegenerative disorder of middle-aged to old individuals. The pathophysiological process of AD is believed to begin many years before the emergence of clinical symptoms. The important influence of congenital genetic aberrations on the development of AD provides a novel opportunity to initiate prenatal to early postnatal pharmacological treatment to address the role of this critical period of brain development in the disease.

**Methods:**

We investigated for the first time the effect of oral treatment during prenatal to early postnatal development with a neurotrophic compound, P021 (Ac-DGGL^A^G-NH2), on neurobehavior and AD-like pathology in 3xTg-AD, a transgenic mouse model of AD. The transgenic and control wild-type female mice were treated from prenatal day 8 to postnatal day 21 with a custom-made diet containing P021 or a vehicle diet, followed by a standard diet. AD-type cognitive function and pathological features were studied during adulthood and old age.

**Results:**

The P021 treatment rescued cognitive deficits at 4 months, reduced abnormal hyperphosphorylation and accumulation of tau at known major AD neurofibrillary pathology–associated sites, and decreased Aβ plaque load at 22 months in 3xTg-AD mice. Prenatal to early postnatal treatment with P021 also ameliorated certain markers of postsynaptic deficits, including PSD-95 levels and CREB activity, and decreased one measure of neuroinflammation, GFAP level in the brain at 4 and 22 months in 3xTg mice.

**Conclusions:**

These findings suggest that neurotrophic impairment during early development can be one of the etiopathogenic factors of AD and that the neurotrophic peptide mimetic is a potential early prevention strategy for this disease.

## Background

Alzheimer’s disease (AD) is the most common cause of dementia in middle-aged and old individuals, which contributes significantly to health care burden, especially because of the lack of an effective therapy due to its multifactorial and heterogeneous nature and involvement of several different etiopathogenic mechanisms [1, 2]. An estimated 5.7 million Americans suffer from AD; this number includes an estimated 5.5 million people 65 years of age and older, and approximately 200,000 individuals younger than 65 years of age who have the early-onset form of the disease [3]. By 2050, nearly one million new cases are expected to develop per year, and the total estimated prevalence is expected to be 13.8 million [4].

Clinically, AD is characterized by memory deficits, followed by a decline in other cognitive functions [5, 6]. The two major histopathological hallmarks in the brains of patients with AD are extracellular senile plaques consisting of amyloid-β (Aβ) peptides [7] and intracellular neurofibrillary tangles (NFTs) composed of abnormally hyperphosphorylated tau protein [8]. Tau pathology is believed to cause neurodegeneration by disrupting the microtubule network and, consequently, axoplasmic transport [9, 10]. Beta-amyloid plaques are reported to contribute to cell death by interfering with neuron-to-neuron communication at synapses [11]. Various inflammatory processes and cytokines may also have a role in the pathology of AD [12]. Tau and Aβ pathologies occur several years before the onset of clinical symptoms [13, 14]. After disease onset, it becomes increasingly difficult to treat after neurons start to degenerate, and finely tuned neuronal circuits and cognitive skills are not easily recovered at later stages. Thus, the development of drugs for early prevention and treatment is considered necessary research goals.

Currently, the five FDA-approved drugs available for AD treatment (donepezil, galantamine, rivastigmine, memantine, and donepezil/memantine combination) only provide symptomatic benefit, with little effect on the underlying pathology [15, 16]. Obviously, there is urgency to find an effective disease-modifying therapy. Thus, a successful therapeutic strategy for AD may include both inhibition of neurodegeneration and stimulation of regeneration of affected areas of the brain.

In AD-transgenic mice, brain-derived neurotrophic factor (BDNF), when administered after disease onset, can reverse synapse loss, partially normalize aberrant gene expression, improve cell signaling, and restore learning and memory [17, 18]. BDNF exerts substantial protective effects on crucial neuronal circuitry involved in AD, acting through amyloid-independent mechanisms.

During early brain development, neurotrophic factors provide the appropriate brain milieu necessary for all aspects of neural development, including neuronal proliferation, differentiation, growth, and migration [19]. In APP-transgenic mice, BDNF restored the expression of two-thirds of gene sets that were perturbed as a result of mutant APP expression in both the entorhinal cortex and the hippocampus, and learning and memory improved on two separate hippocampus-dependent tasks [17].

Our laboratory generated a 4-mer ciliary neurotrophic factor (CNTF) small molecule peptide mimetic to which adamantylated glycine was added at the C-terminal to generate compound 021 (P021: Ac-DGGL^A^G-NH2), which was found to enhance neuronal proliferation and differentiation by competitively inhibiting leukemia inhibitory factor and by increasing the transcription of BDNF [20–22]. Oral administration of the compound P021 could rescue cognitive aging by enhancing neurogenesis and neuronal plasticity and by decreasing tau levels in aged Fisher rats [20, 21]. Chronic treatment with P021 significantly reduced tau pathology at both the moderate and the severe stages of the pathology in 3xTg-AD mice [23].

The prenatal to early postnatal period is highly susceptible to epigenomic dysregulation with implications for health. Exposure to various environments was shown to induce epigenetic changes and neurodevelopmental deficits and diseases [24]. Stress and hormones, such as glucocorticoids, testosterone, and estradiol, mediate the development of the brain during critical prenatal and early postnatal periods [25]. In a previous study, we found that prenatal to early postnatal treatment with neurotrophic compound P021 rescues developmental delay and at adult age cognitive impairment in a Ts65Dn trisomic Down syndrome mouse model [26]. Maternal choline supplementation and exercise during prenatal period mitigates Alzheimer-like pathology [27, 28].

At present, little information is available on the role of neurotrophins during the early stages of development, which occur during prenatal to early postnatal days (PNDs), on AD later in life. The present study for the first time elucidates the role of prenatal to early postnatal P021 supplementation in preventing the cognitive impairment and major histopathological hallmarks of AD. We found that treatment with P021 from prenatal day 8 till PND 21 prevented cognitive deficits at 4 months, reduced tau and amyloid pathologies at 22 months, and decreased postsynaptic deficits and neuroinflammation in the brain at both 4 and 22 months in 3xTg-AD mice.

## Materials and methods

### Study outline

**Fig. 1:**
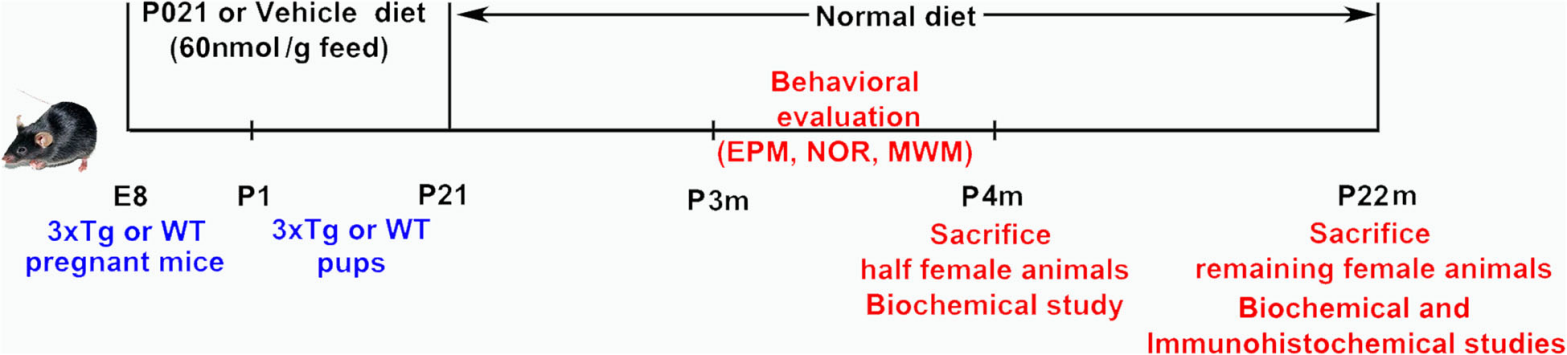
Design of the study. 3xTg-AD or WT mice were treated with P021 or vehicle diet from gestational day 8 till their offspring/pups reached postnatal day 21, at which time the offspring were separated from their mothers and put on normal mouse chow. All of offspring (WT-Veh = 10; WT-P021 = 17; 3xTg-Veh = 12; 3xTg-P021 = 18) were subjected to elevated plus maze, novel object recognition test, and Morris water maze test consecutively from postnatal day 90. Half of the female animals (*n* = 7–8/group) were sacrificed at 4 months (*n* = 4/group) and the remaining animals at 22 months (*n* = 3–4/group) of age. Left cerebral hemisphere homogenate was used for biochemical analysis and right cerebral hemisphere for immunohistochemical. E8, embryonic day 8; P21, postnatal day 21; AD, Alzheimer’s disease; 3xTg, triple-transgenic; WT, wild type; Vh, vehicle diet; P021, peptidergic compound 021 diet

Pregnant 3xTg-AD and wild-type (WT) mice were treated with compound P021 in mouse chow, or as control with vehicle feed from gestational day 8 (E8) till their offspring/pups reached PND21, at which point the offspring were separated from their mother and put on normal mouse chow (Fig. 1). There were four study groups of mice, as follows: offspring of WT treated with vehicle diet (WT-Vh, *n* = 10), offspring of WT treated with P021 diet (WT-P021, *n* = 17), offspring of 3xTg-AD treated with vehicle diet (3xTg-Vh, *n* = 12), and offspring of 3xTg-AD treated with P021 diet (3xTg-P021, *n* = 18). Elevated plus maze, novel object recognition test, and Morris water maze were carried out consecutively from postnatal day 90 with all of mice. About 50% (4 animals/group) of the female offspring (*n* = 7–8 animals/group) were sacrificed at 4 months of age and the remaining 50% (3–4 animals/group) at 22 months of age. Left cerebral hemisphere homogenate was used for biochemical analysis and right cerebral hemisphere for immunohistochemical studies.

### Design and synthesis of P021

The peptidergic compound P021 (Ac-DGGLAG-NH2; mol. wt. of 578.3) corresponds to a biologically active region of human CNTF (amino acid residues 148–151) to which adamantylated glycine was added at the C-terminal to increase its stability and lipophilicity [29, 30]. The peptide was synthesized and purified by reverse-phase high-performance liquid chromatography to ~ 96% purity, and the sequence of the peptide was confirmed by mass spectrometry, as described previously [22].

P021 is quite stable in artificial gastric juice (~ 90% during 30 min) and in artificial intestinal juice (> 95% during 120 min). Blood–brain barrier (BBB) studies on P021, which were carried out through a commercial service (APREDICA, Watertown, MA, USA), demonstrated that enough P021 crossed the BBB to exert its effect in the brain [22].

### Animals and housing

The 3xTg-AD mouse represents one of the most biologically relevant animal models described so far, as it replicates all histopathological and behavioral hallmarks of AD [31]. The 3xTg-AD mice harbor three AD-related genetic loci: human PS1 M146V, human APP_SWE_ K670M/N671L, and human tau P301L. These mice display both plaque and tangle pathologies. Aβ deposition is progressive, with intracellular immunoreactivity detected in some brain regions at as early as 3 to 4 months of age. Extracellular Aβ deposits appear by 6 months of age in the frontal cortex and become more extensive by 12 months of age. Changes in tau occur late; by 12 to 15 months of age, aggregates of conformationally altered and hyperphosphorylated tau are detected in the hippocampus. Cognitive impairments first manifest as a retention/retrieval deficit and not as a learning deficit at 4 months of age and occur prior to the occurrence of plaques and tangles [32].

The homozygous 3xTg-AD mice were obtained from Dr. Frank LaFerla (University of California, Irvine, USA) through the Jackson Laboratory (New Harbor, ME, USA). The background of the 3xTg-AD mice is a hybrid 129/Sv × C57BL/6. The non-transgenic WT mice used were from the same strain and genetic background and were also obtained from Jackson Laboratory. Mice were housed and bred in accordance with approved protocols from our Institutional Animal Care and Use Committee and with the PHS Policy on Human Care and Use of Laboratory animals (revised January 2013).

This study was performed on homozygous 3xTg-AD and WT female mice. Female 3xTg-AD mice were chosen because previous studies demonstrated consistent and overt pathology and worse cognitive performance in female than male 3xTg-AD mice [33–35]. Mice were group-housed (four animals per cage) with a 12:12-h light/dark cycle and with ad libitum access to food and water.

### Treatment of animals with P021

We treated 2- to 3-month-old pregnant 3xTg-AD and WT mothers with compound P021 in mouse chow, or with vehicle feed as control at E8 until their offspring/pups reached PND21, at which point the offspring were separated and put on standard mouse chow (Fig. 1). Treatment was administered as 60 nmol peptide/g formulated diet (Research Diets; New Brunswick, NJ, USA). The vehicle-treated control animals received the same diet but without the peptide. On average, each mouse consumed ~ 2.7 g diet/day.

### Behavioral procedures

#### Elevated plus maze

Elevated plus maze was used to evaluate anxiety/emotionality of the mice. It consisted of four arms (30 × 5 cm) connected by a common 5 × 5 cm center area. Two opposite-facing arms were open (OA), whereas the other two facing arms were enclosed by 20-cm-high walls (CA). The entire plus maze was elevated on a pedestal to a height of 82 cm above floor level in a room separated from the investigator. Ambient luminosity was maintained at 60 lx to control the anxiogenic feature of light for rodents. The mouse was placed onto the central area facing an OA and allowed to explore the maze for a single 8-min session. Between each session, any feces were cleared from the maze, and the maze floor was cleaned with 70% alcohol to remove any urine or scent cues. For each animal, the number of CA entries, OA entries, and amount of time spent in CA and OA were automatically recorded by a video.

#### Novel object recognition test

The novel object recognition memory task has been used to evaluate hippocampus-dependent memory in rodents through an evaluation of the differences in the exploration time of novel and familiar objects [36]. The testing apparatus was a classic open field (i.e., a PVC square arena, 50 × 50 cm, with walls 40 cm high). The open field apparatus was placed in a part of the room separated from the investigator and was surmounted by a video camera connected to a computer. Testing consisted of three different phases: a habituation phase, a sample phase, and a test phase. Following initial exposure, four additional 10-min daily habituation sessions were performed for mice to become familiar with the apparatus (50 × 50 × 40 cm) and the surrounding environment. On the fifth day, every mouse was first subjected to the sample phase, in which two identical objects were placed in a symmetrical position from the center of the arena, and the mouse was allowed to freely explore the objects for 8 min. After a 20-min delay during which the mouse was returned to its home cage, the animal was reintroduced into the arena to perform the test phase. The mouse was then exposed to two objects for another 5 min: a familiar object (previously presented during the sample phase) and a novel object, placed at the same location as during the sample phase. The area of exploration was set to approximately 2 cm around the object. Data collection was performed using a video tracking system (ANY-maze version 4.5 software; Stoelting Co., Wood Dale, IL, USA). The object discrimination index (DI) during the test phase was calculated as DI = [(time spent exploring the new object − time spent exploring the old object)/(time spent exploring both old and new objects)] × 100%.

#### Morris water maze

The Morris water maze task was used to evaluate spatial learning and memory of the mice [37]. A total of 57 mice were subjected to the Morris water maze task at about 4 months of age. The test was performed in a circular white pool (with a diameter of 180 cm and a height of 60 cm) filled with nontoxic white dye–tinted water and maintained at room temperature (20 ± 1 °C). The maze was designed with two virtual principal axes, with each line bisecting the maze perpendicular to the other one to divide the maze into four equal quadrants. The end of each line demarcated four cardinal points: north, south, east, and west. A platform was positioned in the middle of one of the quadrants submerged 1 cm below the water surface.

To test the ability of mice to undergo Morris water maze test, a cued test that requires motor strength, ability to see visual cues, swim, and ability to climb the platform was performed 24 h before the formal experiment. The platform was protruded about 12 cm from the water surface through a wooden pole, and then the mice were gently placed in the water to sail freely. Those mice that could not swim or climb the platform within 90 s were eliminated. This cued trial was done only once for each animal.

The Morris water maze test was comprised of two parts: the place navigation test and the spatial probe test. In the place navigation test, each mouse performed four trials for four consecutive days from semi-random start positions to find the hidden platform [38]. Each trial was terminated as soon as the mouse climbed onto the hidden platform. If a mouse failed to find the platform within 90 s, it was gently guided to the platform. At the end of each trial, the mouse was left on the platform for 20 s, then removed, dried, and returned to its home cage. To examine spatial memory, a spatial probe test was administered 24 h after the last training session. During the probe test, the platform was removed from the pool and the mouse was allowed to swim freely for 1 min.

### Tissue processing

After completion of the behavioral task at postnatal day 120, 50% of the number of female animals (*n* = 4/group) were perfused, and brain tissue was collected for immunohistochemical and biochemical analysis. The remaining female animals continued receiving standard diet and were sacrificed at 22 months of age for immunohistochemical and biochemical analysis.

The animals were anesthetized with an overdose of sodium pentobarbital (125 mg/kg) and transcardially perfused with 0.1 M phosphate-buffered saline (PBS). After perfusion, the brains were removed from the skull immediately. The left hemisphere was dissected into hippocampus, cerebral cortex, cerebellum, and brain stem; immediately frozen on dry ice; and then stored at − 80 °C for biochemical analysis.

The complete right hemisphere was immersion-fixed in 4% paraformaldehyde in 0.1 M PBS for 24–48 h, followed by cryoprotection in a 30% sucrose solution at 4 °C overnight. Later, the 40-μm-thick sagittal sections were cut on a freezing microtome. The sections were stored in glycol anti-freeze solution (ethylene glycol, glycerol, and 0.1 M PBS in 3:3:4 ratio) at − 20 °C until further processing for immunohistochemical staining.

### Immunofluorescence staining and quantification of immunoreactivity

Immunofluorescence staining was performed on free-floating sections, and every 12th brain section was chosen for densitometry and quantification. For immunohistochemical quantification, six brain sections of four animals per group were analyzed. All stainings for fluorescence-intensity quantification were carried out under identical conditions, including all tissue samples for a particular staining processed at the same time, and experimental and control sections photographed under identical settings. The primary antibodies that were used are described in Table 1. The following secondary antibodies were used: Alexa 488-conjugated goat anti-mouse IgG antibody (1:500, Molecular Probes; Carlsbad, CA, USA) and Alexa 488-conjugated goat anti-rabbit IgG antibody (1:500, Molecular Probes).

**Table 1:** Primary antibodies used in this study

Antibody	Specificity	Species	Type	Dilution	Source
43D	Total tau	M	Mono-	0.5 μg/mL WB	Produced in-house
PHF-1	P-tau (Ser396/404)	M	Mono-	1:200 WB 1:500 IF	[39]
AT-8	P-tau (Ser202/T205)	M	Mono-	1:1000 WB	Thermo Fisher Scientific
R134d	Total tau	R	Poly-	1:1000 WB	Produced in-house
GAPDH	GAPDH	R	Poly-	1:1000 WB	Santa Cruz Biotechnology
p-APP	Thr668	R	Poly-	1:1000 WB	Cell Signaling Technology
APP	APP	R	Poly-	1:1000 WB	Generated in our institute
Anti-Aβ	Aβ/APP	R	Mono-	1:200 IF	Cell Signaling Technology
GluR1	GluR1	R	Mono-	1:1000 WB	EMD Millipore
Synaptophysin	Synaptophysin	M	Mono-	1:3000 WB	Millipore
SMI 52	MAP2	M	Mono-	1:4000 WB	Sternberger Monoclonals Inc.
PSD95	PSD95	R	Poly-	1: 1000 WB	Cell Signaling Technology
NR1	NMDAR1	R	Mono-	1:500 WB	ABCAM
GluR 2/3	GluR2 + 3	R	Mono-	1:2000 WB	ABCAM
Synapsin-1	Synapsin 1	R	Poly-	1:40000 WB	Enzo Life Sciences
CREB	CREB	M	Mono-	1:1000 WB	Cell Signaling Technology
p-CREB	Ser133	R	Mono-	1:1000 WB	Cell Signaling Technology
Iba-1	Iba-1	R	Poly-	1:1000 WB	Wako Pure Chemical Industries, Ltd.
SMI22	GFAP	M	Mono-	1:2000 WB 1:1000 IF	Sternberger Monoclonals Inc. (Lutherville, MD,)

*Iba-1* ionized calcium-binding adaptor molecule 1, *GFAP* glial fibrillary acidic protein, *p-tau* phosphorylated tau, *APP* amyloid precursor protein, *Aβ* amyloid-β, *Poly-* polyclonal, *Mono-* monoclonal, *R* rabbit, *M* mouse

### Measurement of Thioflavin-S–positive plaque load

Thioflavin-S–positive (TS+) plaque load was quantified on every 12th section (roughly six sections per set) of four animals from 22-month-old groups. A modified thioflavin-S staining protocol [40] was used as follows. Free-floating brain sections were washed in large volumes of distilled water, incubated in 0.25% KMnO_4_ for 4–5 min, washed with water, and treated with a solution of 1% K_2_S_2_O_5_ and 1% oxalic acid for 40–60 s until the brown color was completely washed out. Sections were then incubated with 0.05% thioflavin-S in water in the dark for 8 min, followed by differentiation in 80% ethanol twice for 1 min each. Sections were then washed three times in distilled water for 1 min each and mounted by using Fluorgel mounting medium and cover slips (Electron Microscopy Sciences; Hatfield, PA, USA). The images were taken by using a Nikon 90i fluorescent microscope, threshold using ImageJ (v.1.46r), and TS+ plaque load was quantified in the hippocampus CA1 and subiculum areas.

### Western blots

The tissue from the left cerebral hemisphere from each mouse stored at− 80 °C was homogenized in a Teflon-glass homogenizer to generate 10% (w/v) homogenate. The pre-chilled homogenization buffer contained 50 mM Tris–HCl (pH 7.4), 8.5% sucrose, 2 mM EDTA, 2 mM EGTA, 10 mM β-mercaptoethanol plus the following protease and phosphatase inhibitors: 0.5 mM AEBSF, 10 μg/mL aprotinin, 10 μg/mL leupeptin, 4 μg/mL pepstatin, 5 mM benzamidine, 20 mM β-glycerophosphate, 50 mM sodium fluoride, 1 mM sodium orthovanadate, and 100 nM okadaic acid. Each homogenate was boiled in 2× Laemmli sample buffer for 5 min, and protein concentration was measured by PierceTM 660-nm protein assay (Thermo Scientific; Rockford, IL, USA).

The samples were subjected to 10% SDS-PAGE and electro-transferred onto Immobilon-P membranes (EMD Millipore; Billerica, MA, USA). The blots were then probed with primary antibodies (Table 1) and developed with the corresponding HRP-conjugated secondary antibody and enhanced chemiluminescence (Pierce Biotechnology, Rockford, IL, USA). Densitometric quantification of protein bands in Western blots was analyzed using Multi Gauge version 3.0 software (FUJIFILM North America; Valhalla, NY, USA).

### Statistical analysis

The statistical analyses were conducted using SPSS version 17.0 (© SPSS Inc., 1989–2007; Chicago, IL, USA), SASv5 software (SAS Institute; Cary, NC, USA), and GraphPad Prism version 8.0 (GraphPad Software Inc.; La Jolla, CA, USA). Data are presented as mean ± S.E.M. The normality of the data was determined by using the Kolmogorov–Smirnov test. For analysis involving multiple groups, three-way or two-way ANOVA followed by Tukey’s multiple comparisons test was employed. Further intergroup comparisons were also performed using unpaired two-tailed *t* tests. For all purposes, *p* < 0.05 was considered as statistically significant.

## Results

### Prenatal to early postnatal treatment with P021 prevents cognitive deficits at ~ 4 months in 3xTg-AD mice

In our previous studies, we found that P021 treatment for different periods at young or old age can rescue the cognitive impairment in mice and rats, and both tau and Aβ pathologies in 3xTg-AD mice [20, 23]. The fact that familial cases of AD, which are caused by certain mutations in APP or presenilin 1 or 2, as well as transgenic mouse models overexpressing these transgenes do not show cognitive impairment and overt pathology till late in adult life led us to investigate the effect of P021 treatment during early development on cognition and AD pathology at adult age in 3xTg-AD mice. We treated timed pregnant mice at E8 till pups born to them reached PND 21 with P021 in mouse chow or as control vehicle only, followed by standard mouse chow (Fig. 1). At ~ 4 months, we studied these animals for any changes in their behavior and pathology.

**Fig. 2:**
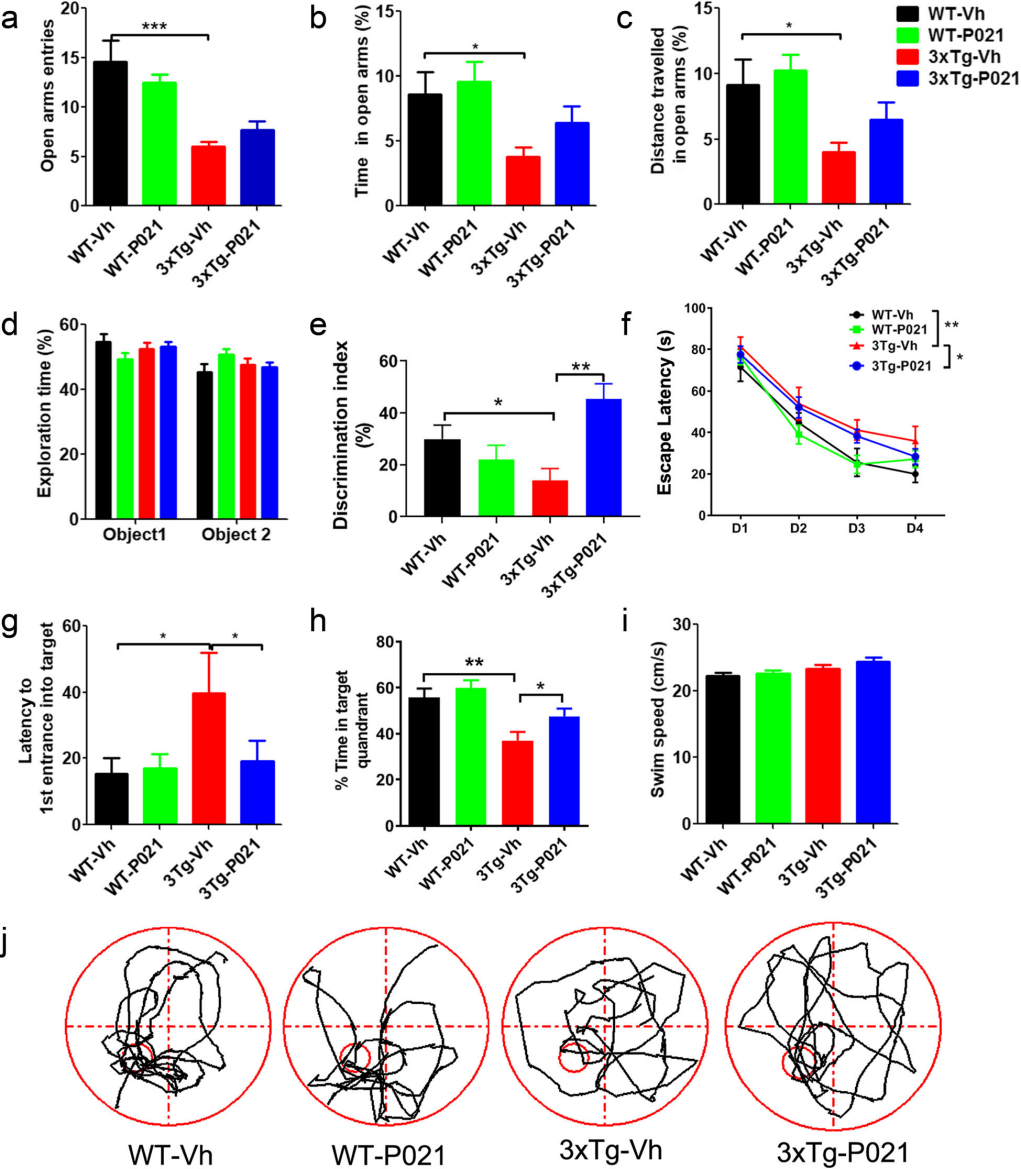
P021 treatment from embryonic day 8 through postnatal day 21 prevents the memory deficits at the age of 3–4 months in 3xTg-AD mice. Elevated plus maze (**a**–**c**), novel object recognition test (**d**, **e**), and Morris water maze (**f**–**j**) were carried out from day 90 to 120. Decrease in 3xTg-Vh mice but not in 3xTg-P021 mice of the number of arm entries (**a**) (two-way ANOVA, interaction *F* (1, 50) = 2.730 *P* = 0.1048; treatment *F* (1, 50) = 0.03329 *P* = 0.8560; genotype *F* (1, 50) = 33.15 *P* < 0.0001); **b** time spent in open arms (two-way ANOVA, interaction *F* (1, 52) = 0.3033 *P* = 0.5842; treatment *F* (1, 52) = 1.482 *P* = 0.2289; genotype *F* (1, 52) = 7.482 *P* = 0.0085); and **c** distance traveled in open arms (two-way ANOVA, interaction *F* (1, 51) = 4.500 *P* = 0.0688; treatment *F* (1, 51) = 0.6570 *P* = 0.4214; genotype *F* (1, 51) = 30.78 *P* < 0.0001). **d** No difference was observed in the percentage of time spent in exploring two identical objects during the 8-min sample phase (three-way ANOVA, genotypes × treatments × objects). **e** A 5-min test phase was carried out 20 min later, and the less-explored object during the sample phase was exchanged with a novel object. Discrimination index (time spent exploring the new object − time spent exploring the old object)/(time spent exploring both old and new objects) × 100% in the test phase was improved in 3xTg-P021 mice (two-way ANOVA, interaction, *F* (1, 42) = 17.46, *P* = 0.0001; treatment, *F* (1, 42) = 3.583, *P* = 0.0653; genotype, *F* (1, 42) = 0.04287, *P* = 0.8370; WT-Vh vs 3xTg-Vh *P* = 0.0483; WT-Vh vs WT-P021 *P* = 0.3703; 3xTg-Vh vs 3xTg-P021 *P* = 0.0007). **f** During the acquisition phase for 4 days, the escape latency to reach the hidden platform was increased in 3xTg-AD mice compared with WT controls, and treatment with P021 did not prevent it as determined by the three-way ANOVA but showed a clear tendency as determined by the two-tailed *t* test (three-way ANOVA, days, *F* (3, 192) = 91.77, *P* < 0.0001; genotype, *F* (1, 192) = 14.86, *P* = 0.0002; treatment, *F* (1, 192) = 2.596, *P* = 0.1088; two-tailed *t* tests, WT-Vh vs 3Tg-Vh, *P* < 0.01; 3xTg-Vh vs 3xTg-P021, *P* < 0.05). **g** In the probe trial, 3xTg-AD mice took more time in the first entrance into the target zone, and treatment with P021 decreased the latency significantly (two-way ANOVA, interaction *F* (1, 59) = 6.324 *P* = 0.0147; treatment *F* (1, 59) = 3.634 *P* = 0.0615; genotype *F* (1, 59) = 4.601 *P* = 0.0361; WT-Vh vs 3xTg-Vh *P* = 0.0095; WT-Vh vs WT-P021 *P* = 0.9737; 3xTg-Vh vs 3xTg-P021 *P* = 0.0132). **h** 3xTg-AD but not 3xTg-AD-P021 mice spent less time in the target quadrant than did WT mice (two-way ANOVA, interaction *F* (1, 56) = 0.8808 *P* = 0.3520; treatment *F* (1, 56) = 6.928 *P* = 0.0109; genotype *F* (1, 56) = 22.64 *P* < 0.0001; WT-Vh vs 3xTg-Vh *P* = 0.0007; WT-Vh vs WT-P021 *P* = 0.6163; 3xTg-Vh vs 3xTg-P021 *P* = 0.0745; two-tailed *t* tests, 3xTg-Vh vs 3xTg-P021 *P* = 0.0493). **i** 3xTg-AD mice and WT controls displayed similar swim speed. **j** Cued trial, examples of swim path to the cued platform; all animals found the cued platform. *n* = 10 for WT-Vh; *n* = 17 for WT-P021; *n* = 12 for 3xTg-Vh and *n* = 18 for 3xTg-P021. *P < 0.05, **P < 0.01, ***P < 0.001

Anxiety-like behavioral changes are reported to occur in the elevated plus maze task: OA entries, time in OAs, and distance traveled in OAs in AD mice. In this study, we found that in the elevated plus maze test, OA entries, time in OAs, and distance traveled were all significantly decreased in 3xTg-Veh but not in 3xTg-P021 mice. These results suggest that P021 did not relieve anxiety-like behavior in the elevated plus maze task in 3xTg-AD mice (Fig. 2a–c).

To examine whether treatment with P021 could rescue the short-term memory impairment in 3xTg-AD mice, we conducted a one-trial novel object recognition task with a 20-min interval between the sample phase and the test phase. In the one-trial novel object recognition task, each group of mice spent equal amounts of time exploring the two identical objects during the 8-min sample phase (Fig. 2d). After 20 min, the less-explored object during the sample phase was replaced with a novel object during a 5-min test phase. Compared to WT mice, 3xTg-AD mice spent less time exploring the novel object, whereas 3xTg-P021 mice spent a longer time exploring the novel object than did 3xTg-Vh mice (Fig. 2e). These results indicate short-term memory impairment in 3xTg-Vh and its prevention in 3xTg-P021 animals.

To investigate whether treatment with P021 during early development can prevent spatial memory impairment in 3xTg-AD mice, the Morris water maze task was conducted. 3xTg-Veh mice showed a noticeable increase in the escape latency during the training days (Fig. 2f), and they also took more time on the first entrance to the target platform and spent less time in the target quadrant than WT mice, whereas in 3xTg-P021 mice, the latency to the first entrance into the target platform was reduced, and the time spent in the target quadrant was similar to that in the WT-Vh mice (Fig. 2g, h). There was no significant difference in the swim speed (Fig. 2i) or cued trial (Fig. 2j) among different groups. Altogether, these results suggest a beneficial effect of P021 treatment during early development in preventing certain measures of cognitive impairment in 3xTg-Vh mice.

### Prenatal to early postnatal treatment with P021 prevents tau pathology at 22 months in 3xTg-AD mice

**Fig. 3:**
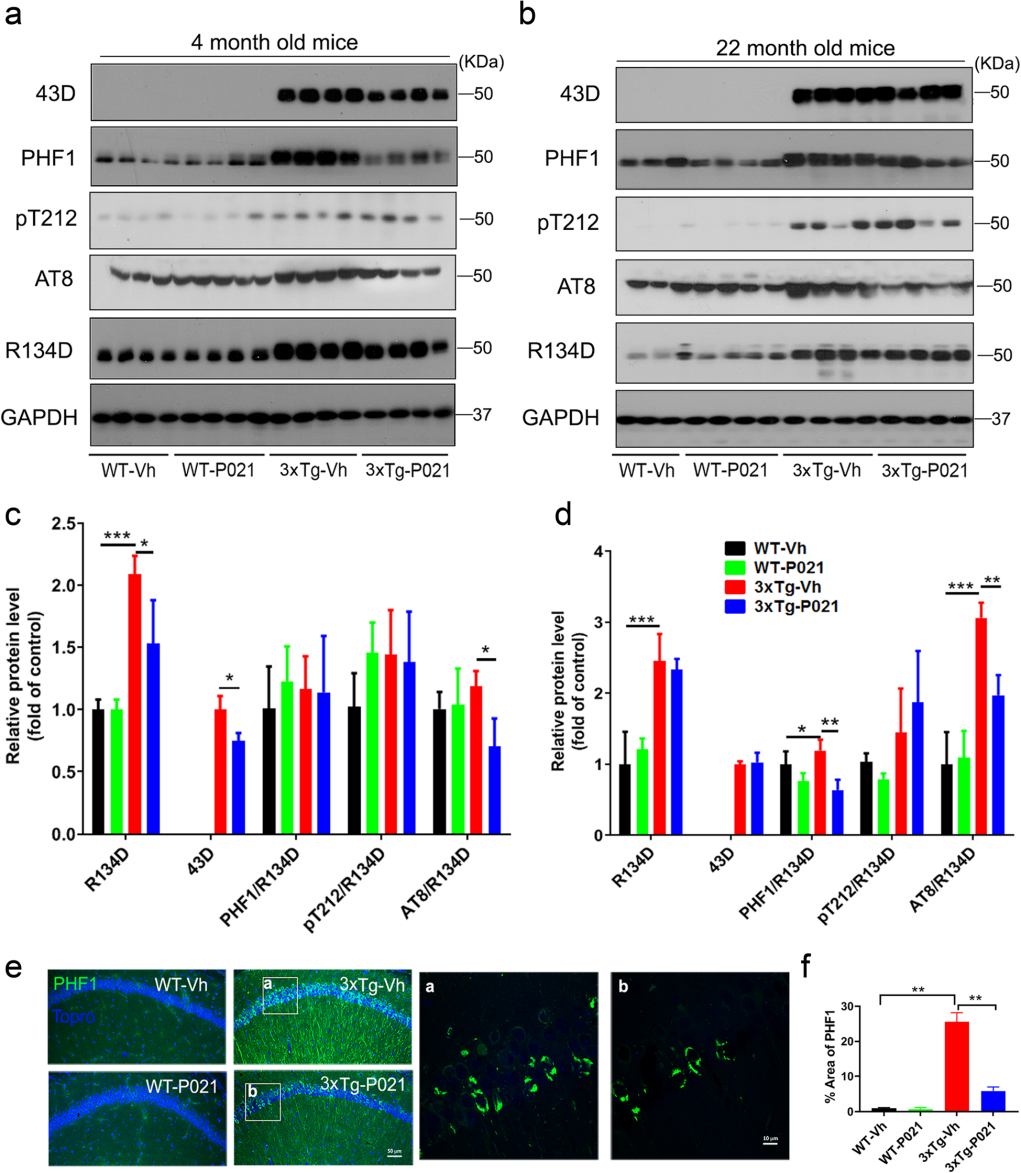
Prenatal to early postnatal treatment with P021 prevents Tau pathology at 22 months of age in 3xTg-AD mice. **a**, **b** Western blot analysis of tau pathology in 4- and 22-month-old mice. **c**, **d** Densitometric quantification of blots after normalization with GAPDH or rabbit polyclonal antibody to total tau, R134D. P021 treatment reduced total tau (R134d) and human tau (43D); there was no increase in hyperphosphorylation of tau in 4-month-old 3xTg-AD mice as compared to the WT control but P021 treatment reduced the level of phosphorylation at AT8 site in the former (**a**, **c**). The level of hyperphosphorylation of tau at PHF1 and AT8 sites was increased in the hippocampus and the P021 treatment prevented it at 22 months in 3xTg-AD mice (**b**, **d**). **e** Immunostaining with PHF1 showed a decrease in CA1 area of the hippocampus in P021-treated 3xTg mice at age 22 months (a, b: the high magnification). **f** Analysis of percent area of PHF1 staining. Data were analyzed by two-way ANOVA followed by Tukey’s multiple comparisons test. Further intergroup comparisons were performed using unpaired, two-tailed *t* tests. At each age, *n* = 3–4 for WT-Vh, *n* = 4 for WT-P021, *n* = 4 for 3xTg-Vh, and *n* = 4 for 3xTg-P021. *P < 0.05, **P < 0.01, ***P < 0.001

To determine the effect of treatment with P021 during early development on tau pathology in the hippocampus of 3xTg mice at different ages, we performed Western blots and immunohistofluorescence staining to study changes in the levels and expressions of total and hyperphosphorylated taus. We found that compared with age-matched WT-Vh mice, 4-month-old 3xTg-Vh mice exhibited a significant increase in total tau detected with antibody R134d. P021 treatment decreased the total tau detected with R134d and human-specific tau with antibody 43D (Fig. 3A, C). There was no increase in hyperphosphorylation of tau in 4-month-old 3xTg-Vh mice as compared to the WT control mice. Nevertheless, the P021 treatment was found to decrease the level of tau hyperphosphorylation at Ser202/Thr205 detected with antibody AT8 in 4-month-old 3xTg-AD mice.

In 22-month-old mice, we found an increase in total tau level with antibody R134d and an increase in tau hyperphosphorylation at Ser-396/404 detected with antibody PHF-1, and at Ser202/Thr205 detected with antibody AT8 in 3xTg-Vh compared with WT-Vh. P021 treatment decreased tau hyperphosphorylation both at PHF-1 and AT8 sites, whereas there was only an insignificant decrease in total tau in P021-treated animals; levels of human tau detected with antibody 43D and hyperphosphorylated tau detected with antibody pT212, however, did not change significantly (Fig. 3B, D). To further evaluate changes in hyperphosphorylated tau in 22-month-old mice, we performed PHF-1 immunofluorescence and found a significant increase in the hippocampal CA1 region of 3xTg-Vh mice, whereas it was attenuated in 3xTg-AD-P021 mice (Fig. 3E, F). Altogether, the above data indicate that prenatal to early postnatal treatment with P021 can prevent tau pathology in the hippocampus of 3xTg mice at 22 months of age.

### Prenatal to early postnatal treatment with P021 reduces amyloid plaques and Aβ/APP levels at 22 months in 3xTg mice

**Fig. 4:**
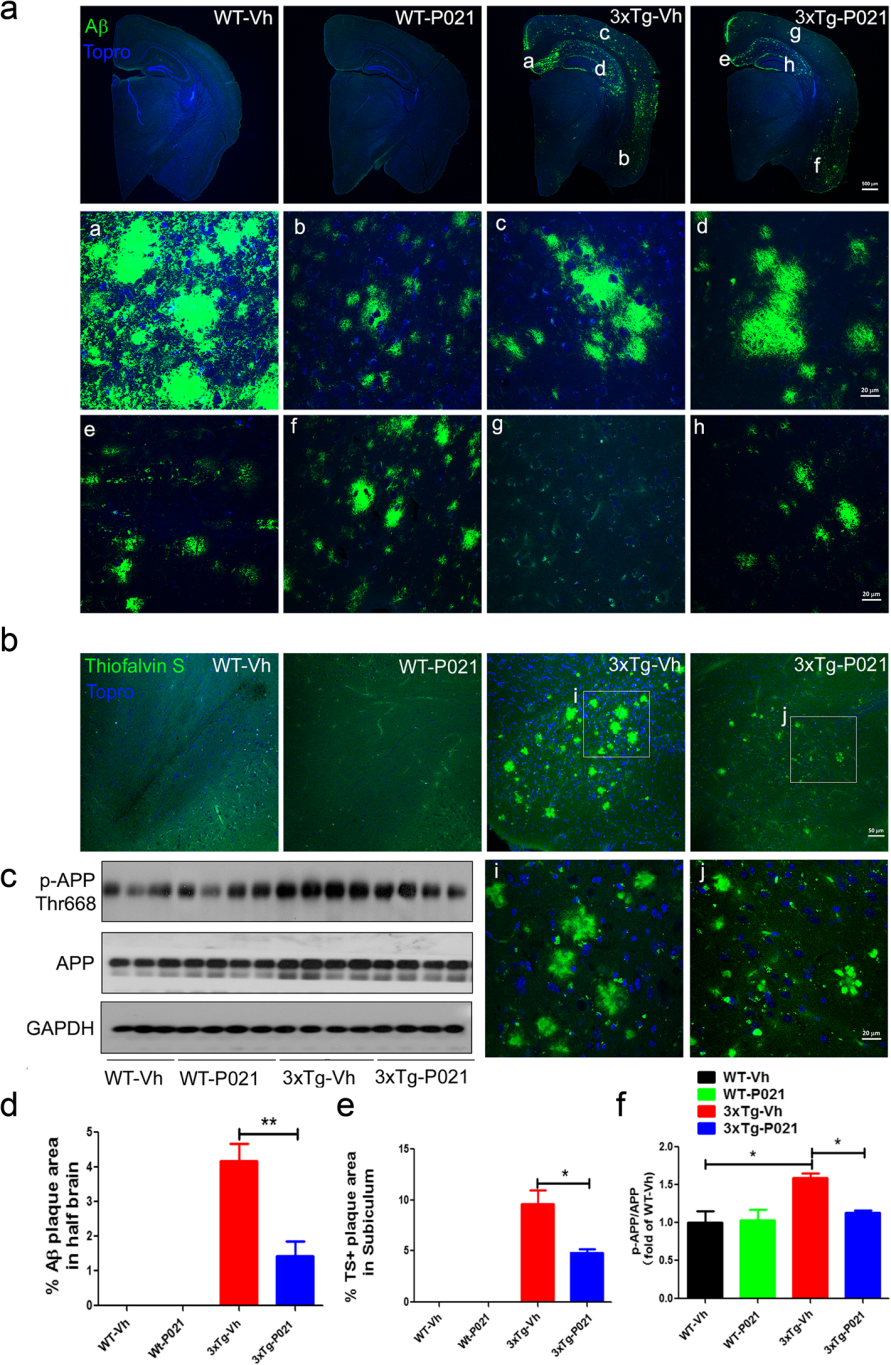
Prenatal to early postnatal treatment with P021 prevents Aβ pathology in 22-month-old 3xTg-AD mice. **a** Aβ immunofluorescence in the coronal brain sections of one half of the brain (a–h: the high magnification, a and e for the subiculum, b and f for the piriform cortex, c and g for the forebrain, d and h for the hippocampus). **b** Thioflavin-S staining in the subiculum region in 3xTg-AD mice (i and j: the high magnification for the subiculum). **c** Representative Western blots of the hippocampus developed with antibodies to p-APP or APP. Quantification of the Aβ-positive staining area in one half of the brain (6 sections per mouse, *n* = 4 mice). **d** Amyloid plaque area seen by immunofluorescence in one half of the brain. **e** Amyloid plaque area detected by thioflavin-S staining in the subiculum (6 sections per mouse, *n* = 4 mice). **f** Relative level of phosphorylation of APP at Thr 668 after normalization with GAPDH (*n* = 4). Data were analyzed by two-way ANOVA followed by Tukey’s multiple comparisons test. Further intergroup comparisons were performed using unpaired, two-tailed *t* tests. *P < 0.05, **P < 0.01

Amyloid plaques are developed in 3xTg-AD mice starting at around 6–9 months of age, and they are first observed in the cerebral cortex and progress to the hippocampus with age [31]. To evaluate the effect of P021 on Aβ pathology at 22 months of age in 3xTg mice, we performed Aβ immunofluorescence in the serial coronal sections of one half of the brain dissected through the midline. Amyloid plaques detected by Aβ antibody were found to be widely distributed in the subiculum, piriform cortex, forebrain, and hippocampus of 3xTg mice (Fig. 4A, a–d), and the plaques in the same regions were markedly reduced in 3xTg-AD-P021 mice (Fig. 4A, e–h), whereas no plaques were observed in the brains of age-matched WT mice. Moreover, the plaque-occupied area was significantly decreased in 3xTg-P021 mice (Fig. 4D). To confirm the changes in Aβ plaque load, we also performed thioflavin-S staining and found that the plaque burden increased predominately in the subiculum of 3xTg-Vh mice, whereas 3xTg-P021 mice showed a significant decrease (Fig. 4B, E). Correspondingly, Western blots showed that the level of phosphorylation of APP at Thr668 was elevated significantly in the hippocampus of 3xTg-Vh mice, and it was rescued in 3xTg-P021 animals (Fig. 4c, f). Collectively, these results revealed that P021 treatment during early development could prevent Aβ plaque pathology during old age in 3xTg mice.

### Prenatal to early postnatal treatment with P021 ameliorates the neuroplasticity related glutamate receptor and postsynaptic deficits and increases CREB activity in 3xTg-AD mice

**Fig. 5:**
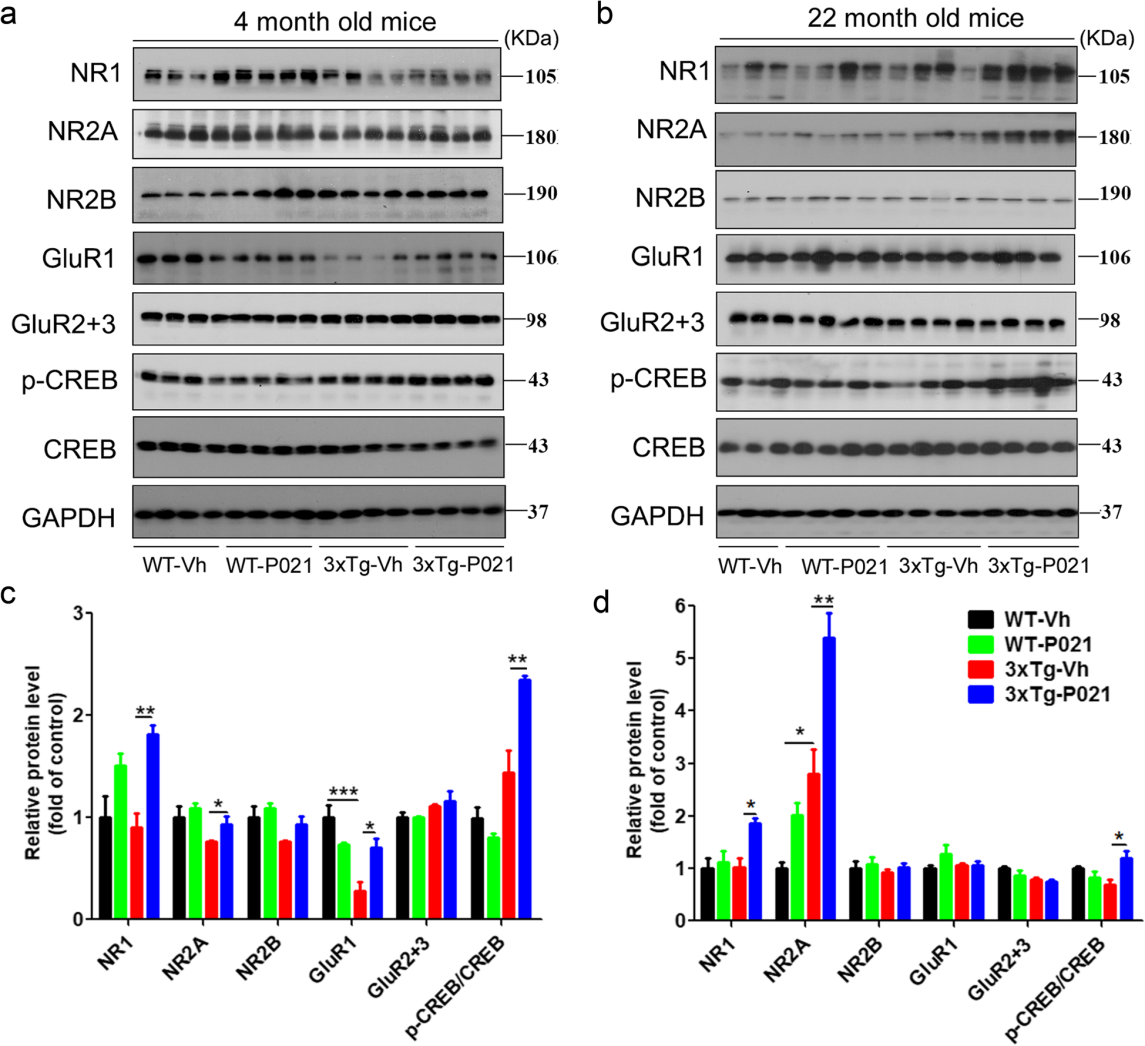
Prenatal to early postnatal treatment with P021 prevents dendritic deficits and increases CREB activity at 4 months and 22 months of age in 3xTg-AD mice. **a**, **b** Representative Western blots of the hippocampus for NR1, NR2A, NR2B, GluR1, GluR2&3, p-CREB, CREB, and GAPDH at 4 months and 22 months in P021- or vehicle-treated mice. **c**, **d** Densitometric quantification of the above proteins after normalization with GAPDH. 3xTg-Vh mice showed a marked decrease of GluR1, while P021 treatment increased the levels of NR1, NR2A, GuR1, and p-CREB significantly at 4 months of age. At 22 months, P021 elevated the levels of NR1, NR2A, and p-CREB in 3xTg-AD mice. **d** Data were analyzed by two-way ANOVA followed by Tukey’s multiple comparisons test. Further intergroup comparisons were performed using unpaired, two-tailed *t* test. *n* = 3–4 animals/group. *P < 0.05, **P < 0.01, ***P < 0.001

Synaptic deficit is a feature of AD pathology. The glutamate receptors AMPA (α-amino-3-hydroxy-5-methyl-4-isoxazole propionic acid) and NMDA (*N*-methyl-d-aspartate) receptors play essential roles in synaptic transmission and long-term potentiation (LTP) as well as cellular mechanisms that are associated with learning and memory [41, 42]. Our previous studies showed that synaptic deficit could be rescued by chronic P021 treatment in adult 3xTg-AD mice [43, 44]. Herein we examined whether the synaptic changes at adult age could be affected by treatment with P021 during a period of early development. At 4 months of age, the level of GluR1 was significantly reduced in 3xTg-Vh mice compared with WT-Vh mice, and after P021 treatment, not only GluR1 but also levels of NR1, NR2A, and phosphorylated CREB (p-CREB) were increased, whereas no significant changes in NR2B and GluR2&3 were found among groups (Fig. 5a, c). At 22 months, we found that levels of NR1, NR2A, and p-CREB were increased in the hippocampus of 3xTg mice with P021 treatment, but no significant changes in NR2B, GluR1, GluR2, and 3 were observed among these groups (Fig. 5b, d).

**Fig. 6:**
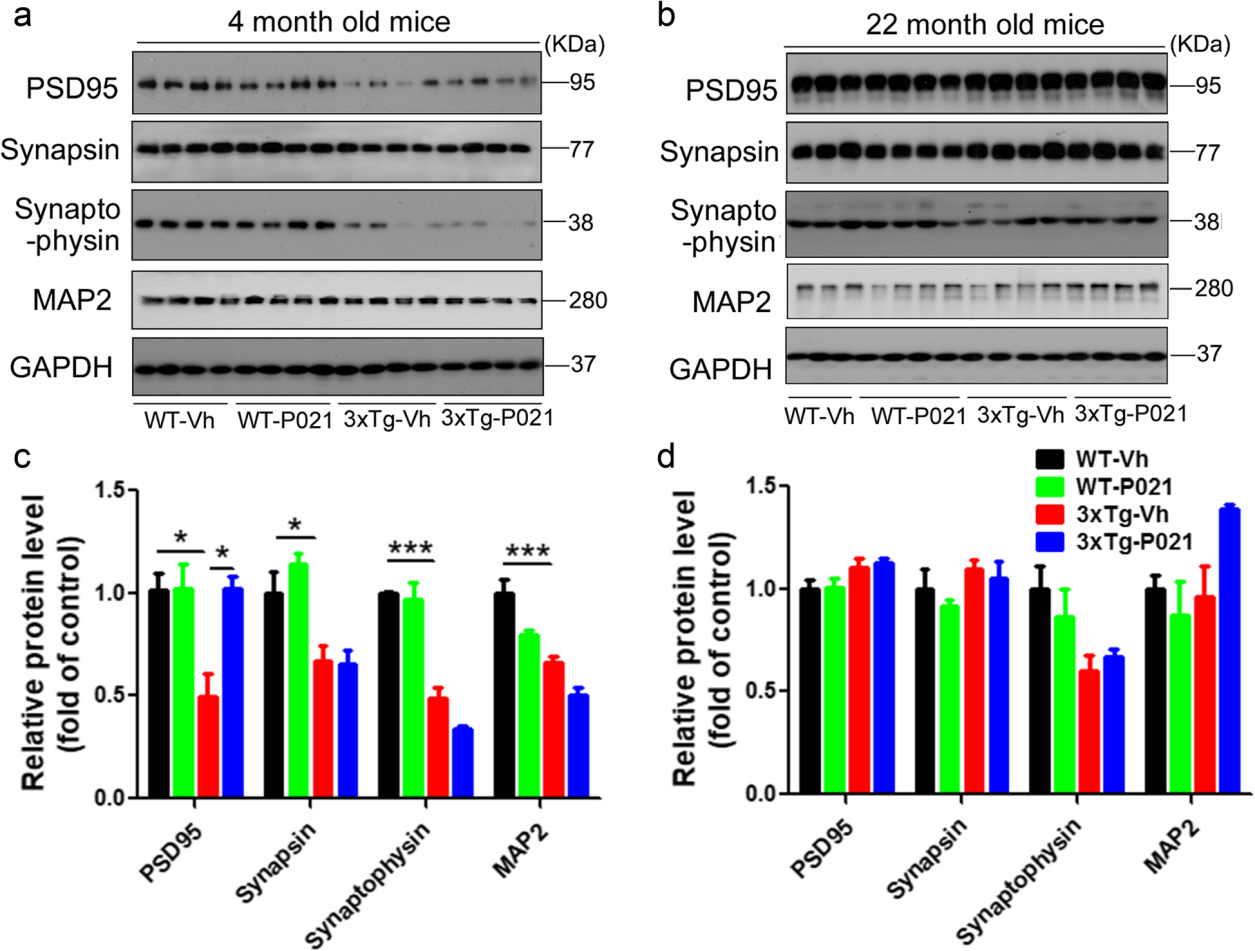
Prenatal to early postnatal treatment with P021 prevents postsynaptic deficit at 4 months of age in 3xTg-AD mice. **a**, **b** Representative Western blots of the hippocampus for PSD95, synapsin, synaptophysin, MAP2, and GAPDH in 4- and 22-month-old mice. **c**, **d** Densitometric quantification of the above proteins after normalization with GAPDH. At 4 months, 3xTg-Vh mice showed a marked decrease in levels of PSD95, synapsin, synaptophysin, and MAP2, while P021 prevented PSD95 deficit (**a**, **c**). No significant change was found in 22-month-old mice (**b**, **d**). Data were analyzed by two-way ANOVA followed by Tukey’s multiple comparisons test. Further intergroup comparisons were performed using unpaired, two-tailed *t* test. *n* = 3–4 animals/group. *P < 0.05, **P < 0.01, ***P < 0.001

Synaptic loss is known to be correlated with cognitive decline in AD [45, 46]. We therefore studied whether treatment with P021 during early development can ameliorate deficits in markers of dendritic and synaptic plasticity in 3xTg-AD mice at different stages of the disease. We found that levels of PSD95, synapsin, synaptophysin, and MAP2 were all notably decreased in 3xTg-Vh mice, but only the PSD 95 deficit was rescued in 3xTg-P021 animals, suggesting an important role of PSD 95 in cognitive function in 3xTg mice (Fig. 6a, c). While no significant changes in levels of PSD95, synapsin, synaptophysin, and MAP2 were found between 3xTg and WT mice at 22 months of age, there was a trend for an increase in these levels in 3xTg-P021 mice (Fig. 6b, d).

### Prenatal to early postnatal treatment with P021 prevents neuroinflammation at 4 and 22 months in 3xTg mice

**Fig. 7:**
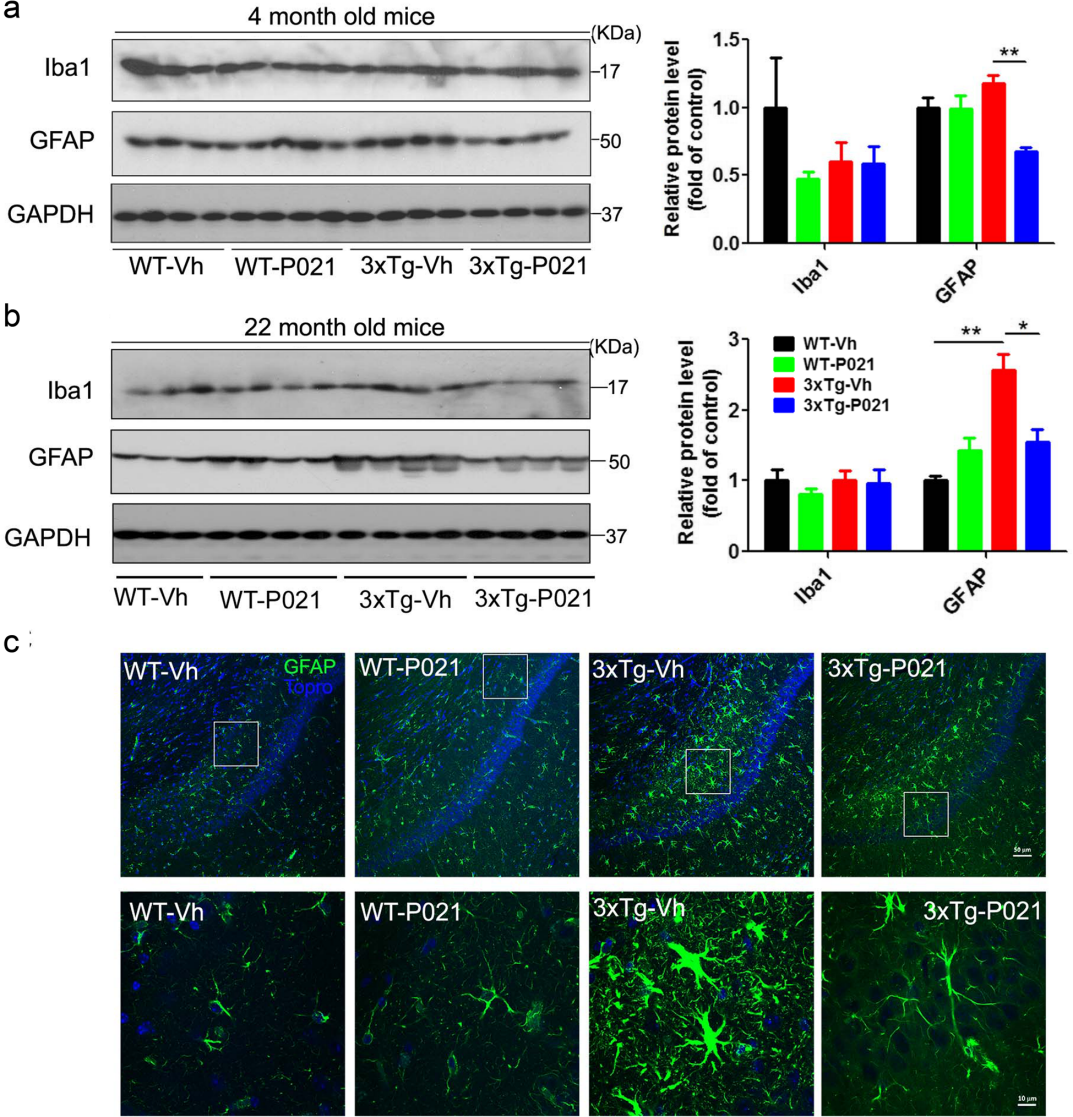
Prenatal to early postnatal treatment with P021 prevents neuroinflammation at 4 and 22 months of age in 3xTg-AD mice. Representative Western blots developed with Iba1 and GFAP antibodies and their quantification after normalization with GAPDH in the hippocampus of 4-month- (**a**) and 22-month-old mice (**b**). 3xTg-Vh mice showed a marked increase of GFAP at both 4 months and 22 months, and treatment with P021 significantly decreased the GFAP level, while no significant change in the level of Iba1 was found in either 4- or 22-month-old mice. **c** Representative image of GFAP immunostaining in CA1 of 22-month-old 3xTg-AD mice; a marked increase in the intensity of GFAP staining, larger cell body, and short processes were observed, while P021 attenuated these changes. Data were analyzed by two-way ANOVA followed by Tukey’s multiple comparisons test. Further intergroup comparisons were performed using unpaired, two-tailed *t* tests. *n* = 3–4 animals/group. *P < 0.05, **P < 0.01

Microglia and astrocytes are the most abundant cell types in the brain and are instrumental in supporting neuronal health and function. However, microgliosis and astrogliosis can also be major contributors to chronic neuroinflammation that diminishes neuronal integrity, and they contribute to neurodegeneration in tauopathies [47, 48]. Glial fibrillary acidic protein (GFAP) plays an important role in brain inflammation and in mediating astrocyte-neuron interactions [49]. In a previous study, we found that P021 can stimulate the expression of GFAP [20]. In the present study, we investigated expressions of Iba-1 (a microglial marker) and GFAP in the hippocampus by Western blots and immunofluorescence. We detected no significant changes in expression of Iba-1 among groups at both 4 and 22 months (Fig. 7a, b), whereas GFAP was significantly increased in the 3xTg-Vh mice at 4 and 22 months, and this increase was rescued in 3xTg-P021 animals (Fig. 7a, b). Additionally, GFAP immunoreactivity was also highly elevated in the CA1 region of 3xTg-Vh mice and was rescued in 3xTg-P021 animals (Fig. 7c, upper panel). Moreover, at high magnification, astrocytes in 3xTg-Vh mice were amoeboid and showed large cell body and shorter processes (an advanced activated phenotype), whereas astrocytes in 3xTg-P021 mice appeared ramified with long processes and small cell body (resting phenotype) (Fig. 7c, lower panel). These findings suggested that treatment with P021 during early development can prevent certain components of neuroinflammation involving astrocytes in the hippocampus of 3xTg mice.

## Discussion

The present study shows for the first time that prenatal to early postnatal treatment with the neurotrophic compound P021 can prevent not only cognitive deficit but also AD-type pathological changes, which include tau and Aβ pathologies, postsynaptic deficit, and neuroinflammation later in life in 3xTg mice. These findings suggest the novel concept that AD can be a developmental disability with a late-life phenotype and that adjusting the brain molecular milieu by providing appropriate neurotrophic support at a critical period of brain development can be an effective therapeutic strategy for the prevention of AD and related conditions later in life.

Neural tube formation occurs approximately mid-gestation in rodents, on E 10.5–11 and 9–9.5 in rats and mice, respectively, with birth typically occurring on E20–21 [50, 51]. The mouse cortex reaches approximately 90% of its adult weight by postnatal day 20, the typical age of weaning in rodents. In humans, brain weight reaches a similar plateau by 2–3 years of age. Thus, based on brain weight alone, postnatal day 21 in mice appears to correspond to 2- to 3-year-old humans [52]. The expression of pro-BDNF peaks on postnatal day 15 and declines in later stages, whereas the expression of mature BDNF peaks on postnatal day 21 and stays high in adulthood in mice [53]. Although there is no evidence that neurotrophic factors are reduced during early brain development in AD, BDNF deficit has been reported in Down syndrome during early development [54, 55]; Down syndrome is a developmental disability that develops AD pathology during the fourth decade of life [56]. We hypothesized that disease-causing mutations in APP, PS1, and tau could affect the neurotrophic milieu during the early development period. Toward this hypothesis, we treated the timed pregnant 3xTg-AD mice at E8 until the pups that were born to them reached PND 21 with P021 in mouse chow. Thus, pups received P021 through the placenta till birth and then from mother’s milk till weaning and separation from the mother on PND 21; around PND 15–18, pups also start nibbling on chow that is dropped in the cage.

A central objective of the field of dementia research is to inhibit, or to at least effectively modify, the course of AD, but at present, no effective treatment or cure for AD is available. CNTF is a pluripotent neurotrophic factor that has a potent effect on the development and maintenance of the nervous system, inducing neuronal survival and differentiation by stimulating gene expression of sensory, sympathetic, and motor neurons [57]. However, the clinical therapeutic usage of recombinant neurotrophic factors is limited because of the insurmountable hurdles of unfavorable pharmacokinetic properties, poor BBB permeability, and severe adverse effects [58]. P021, which was generated in our laboratory, is a small water-soluble compound, is BBB-permeable, has suitable pharmacokinetic properties for oral administration, and does not induce the systemic adverse effects associated with recombinant CNTF or BDNF [21, 23, 59]. P021 was shown to rescue cognitive impairment in rodent models of AD via increased BDNF expression [20, 23, 54]. Treatment with P021 during prenatal till early postnatal development was found to rescue developmental delay and AD-like hippocampus-dependent cognitive impairments in adult mice with Down syndrome [26]. In the present study, P021 administered orally to mouse mothers during gestation and weaning of their offspring apparently reached significant amounts in the offspring’s brain to exert beneficial effect on cognitive performance, probably via increased NR1, NR2A, and CREB expression, by rescue of the PSD95 deficit and prevention of tau and Aβ pathologies.

AD is a pathological process that starts in mid-life but remains undetected until it causes dementia, at which time a clinical diagnosis of AD dementia can be made. Recent revisions to diagnostic criteria define three stages of AD, namely preclinical AD [60], mild cognitive impairment due to AD (MCI-AD) [61], and AD dementia [62]. Meanwhile, the 3 × Tg-AD mouse model manifests cognitive impairment at 4 months as a deficit in long-term retention, but develops Aβ accumulation at approximately 6–9 months of age, and tau pathology starting at a slightly later age [29, 63]. Because AD patients as well as transgenic mouse models of AD have a long preclinical stage that can last for several decades in humans, it is meaningful to investigate the preventive effect of intervening at the earliest stages of AD. The present study shows that prenatal to early postnatal treatment with P021 can significantly prevent both tau and Aβ plaque pathologies in adult 3xTg mice. Previously, we showed that P021 increases the transcription and expression of BDNF in mice and rats [1, 20, 23, 26, 44, 58]. The beneficial therapeutic effect of P021 apparently involves the increase in the expression of BDNF, which via the TrkB-PI3K-AKT pathway leads to inhibitory phosphorylation of GSK3β at Ser9 [23]. And the inhibition of GSK3β in turn prevents abnormal hyperphosphorylation of tau at several major sites and through reduction of phosphorylation of APP at Thr 668 its amyloidogenic processing and Aβ pathology [58].

AD has been characterized as a synaptic failure [64]. Synapse loss is an early feature of AD, and there is a strong correlation between the severity of dementia and the extent of synaptic loss. Accordingly, it has been proposed that synaptic loss underlies the memory impairment evident in the early phase of AD and that since plasticity is important for neuronal viability, persistent disruption of plasticity may account for the frank cell loss typical of later phases of the disease [32, 65, 66]. Synaptic dysfunction, including LTP deficit, occurs prior to overt plaque and tangle pathologies in 3xTg-AD mice [62]. In the present study, we observed a significant reduction in the density of the presynaptic markers synapsin and synaptophysin, dendritic marker MAP2, and postsynaptic marker PSD95 at 4 months. This dendritic and synaptic loss may lead to deficit in synaptic plasticity. We found that treatment with P021 from prenatal to early postnatal development can prevent synaptic loss, as evidenced by rescue of PSD 95 level. These findings led us to speculate that treatment with P021 during early development probably ameliorates the changes in homeostasis of brain milieu and provides an optimal microenvironment for neuronal proliferation and synaptogenesis and thus enhanced neuronal plasticity. Deficits in synaptic markers were found at age 4 months but not at 22-month 3xTg-AD mice. The exact reason for this apparent discrepancy is, at present, not known and will have to be investigated in future studies. Apparently, like in AD, the synaptic degeneration is quite marked and precedes tau and Abeta pathologies in 3xTg-AD mice. At 22 months, we speculate, there are also age-associated losses in synaptic markers and the differences, especially in presynaptic markers between 3xTg-AD and their genetic background-matched control animals, are probably inconsistent and below the detection limit.

Neuroinflammation has been described as an important contributor to AD pathogenesis and cognitive decline [12, 67]. Neuroinflammation is also seen in 3xTg-AD mice [68]. In the present study, we found astrogliosis can be prevented by treatment with P021 during early development in 3xTg-AD mice. Neuroinflammation in 3xTg-AD mice has been shown to involve Aβ and tau pathologies [69]. It is thus possible that treatment with P021 during early development, by virtue of its effect on Aβ and tau pathologies, normalizes the neuroinflammation in 3xTg-AD mice, which in turn contributes to the beneficial effect of P021 on neuronal and synaptic deficits, and cognition.

Although this study provided a proof of principle of the beneficial effect of prenatal to early postnatal treatment with P021 on prevention of cognitive impairment and AD-like pathological changes, it lacks information on the level of the compound that reached the brain in fetuses and pups during various stages of development. A dosing study employing the direct administration of P021 to the pups and analysis of the level of the compound in the brain in fetuses and pups remains to be conducted. Another major limitation of the study is that it did not determine whether the beneficial effect of the P021 treatment is due to this treatment during the prenatal or the postnatal period, or both. It is not known if there is any neurotrophin deficit, whether related to CNTF, BDNF, or other neurotrophins, during development in the AD model used in the present study. Thus, it remains to be investigated in future studies whether the treatment here is making up for a deficit, or alternatively, there is no deficit promoting eventual AD pathology but the further increase in neurotrophin-related mechanisms is nevertheless introducing a protective or preventative function. From the present study, we also not know whether the beneficial effects of P021 are simply through the reduction of tau or Aβ pathologies or neuroinflammation or increase in one or more neuronal plasticity-related mechanisms or having multiple direct effects. The present study used only female AD mice for biochemical studies. Thus, another important information which must await future studies is whether both male and female gender will benefit equally from neurotrophic treatment during the period of early development.

The 3xTg-AD mouse model though faithfully reproduces characteristic features of AD, i.e., cognitive impairment and neurodegeneration associated with Aβ plaques and neurofibrillary pathology of hyperphosphorylated tau, the underlying disease mechanism in this triple-transgenic model which incorporates two APP and one presenilin-1 AD and one tau frontotemporal dementia mutations is probably very different from human AD. However, the beneficial therapeutic effect of P021 we found in previous studies in aged mice and rats, Ts65Dn trisomic Down’s mice, and in a rat model of protein phosphatase inhibition suggests the therapeutic potential of this compound. Double-blind placebo-controlled human clinical trials of this compound for various neurodegenerative conditions will be required to understand its potential for human use.

## Conclusions

The present study shows that prenatal to early postnatal treatment with the neurotrophic compound P021 in 3xTg-AD mice can not only prevent certain cognitive deficits, possibly in part via the increase in the activity of CREB and levels of glutamate receptors and rescue of PSD95 associated synaptic deficit, but also ameliorate selected pathological hallmarks, including Aβ plaque and tau pathologies and astrocyte-related markers of neuroinflammation.

## Data Availability

The datasets generated and/or analyzed during the present study are available from Dr. Wei Wei through the corresponding author upon reasonable request.

## Abbreviations

AD: Alzheimer’s disease
AKT: Protein kinase B
AMPA: α-Amino-3-hydroxy-5-methyl-4-isoxazolepropionic acid
APP: Amyloid precursor protein
Aβ: Amyloid-β
BDNF: Brain-derived neurotrophic factor
CNTF: Ciliary neurotrophic factor
CA: Closed arm
CREB: cAMP response element-binding protein
E: Gestational day
GAPDH: Glyceraldehyde 3-phosphate dehydrogenase
GFAP: Glial fibrillary acidic protein
GluR: Glutamate receptor
LTP: Long-term potentiation
MCI-AD: Mild cognitive impairment due to AD
MWM: Morris water maze
NFTs: Neurofibrillary tangles
NMDA: *N*-Methyl-d-aspartate acid
NR: *N*-Methyl-d-aspartate receptor
NOR: Novel object recognition
OA: Open arm
P021: Peptidergic compound 021, Ac-DGGL^A^G-NH2
PHF: Paired helical filament
PI3Ks: Phosphoinositide 3-kinases
PND: Postnatal day
PSD95: Postsynaptic density protein 95
pS199: Phospho-tau (Ser199)
pT205: Phospho-tau (Thr205)
3xTg: Triple-transgenic
TrkB: Tropomyosin receptor kinase B
TS: Thioflavin-S
WT: Wild type
